# Shame and trauma are critical to understanding the impacts of psychosis: Examining clinical correlates within a tertiary psychosis service cohort

**DOI:** 10.1177/00048674251411085

**Published:** 2026-01-27

**Authors:** Kimberley Davies, Julia M Lappin, Sophie Isobel, Zachary Steel

**Affiliations:** 1 Discipline of Psychiatry and Mental Health, School of Clinical Medicine, Faculty of Medicine & Health, UNSW Sydney, Kensington, NSW, Australia; 2 The Tertiary Referral Service for Psychosis, Prince of Wales Hospital, Randwick, NSW, Australia; 3 Faculty of Medicine and Health, The University of Sydney, Camperdown, NSW, Australia

**Keywords:** Psychosis, psychological distress, schizophrenia, shame, suicide

## Abstract

**Objective:**

This study aimed to examine the association of trauma exposure and shame on the clinical presentation of individuals experiencing psychosis (including suicidal behaviours).

**Methods:**

A retrospective audit of clinical data collected over a 4-year period from a tertiary psychosis service was conducted. All individuals accessing the service had experience of psychosis.

**Findings:**

Data from 201 individuals who completed assessments between 2020 and 2024 were analysed. Exposure to trauma was high, with all reporting experience of at least one traumatic event. Trauma related to psychosis symptoms (64.0%) and treatment experiences following psychosis (57.0%) were particularly prevalent. Exposure to lifespan trauma was positively related to the number of lifetime suicide attempts, *r*(90) = 0.22, *p* = 0.038. Higher levels of shame were associated with an increased frequency of current suicide ideation, External shame: (r(51)= 0.46, *p* < 0.001); Internal shame: (r(50) = 0.45, *p* < 0.001).

**Conclusions:**

These findings highlight different, though related, associations between suicidal behaviours with trauma exposure and shame. While trauma is associated with suicidal behaviours, shame is correlated with suicidal ideation, raising implications for assessment and intervention. Future work could examine whether suicide ideation in this group is influenced by psychological interventions that target shame.

## Introduction

The association between trauma and the development of psychosis is well established (Varese et al., 2012). Recent research suggests that trauma also shapes the trajectory of psychosis, influencing its course and impact over time (Turner et al., 2020). The combined impact of trauma and psychosis is associated with poorer outcomes, including more severe symptoms, heightened emotional distress, increased suicidality, homelessness, victimisation, social dysfunction and disengagement from services (Bailey et al., 2018; Conus et al., 2010; Duhig et al., 2015; Schäfer and Fisher, 2011; Trotta et al., 2015; Turner et al., 2020). Key aspects of the trauma-psychosis relationship nonetheless remain under-explored, particularly the mechanisms by which trauma affects the experience of psychosis. Existing models in the field highlight the role of emotional distress arising from adverse experiences as a contributor to psychosis onset (Fisher et al., 2013; Garety et al., 2001). Among other affective responses to trauma (Grady et al., 2024), shame is commonly elicited, particularly following interpersonal trauma (La Bash and Papa, 2014) and more recent models suggest shame may have a mediatory role (Davies et al., 2025a; Heriot-Maitland et al., 2022; McCarthy-Jones, 2017).

While there is no universally accepted definition of shame, Gilbert’s model is commonly cited where shame is understood as ‘an involuntary response to an awareness that one has lost status and is devalued’ associated with ‘the inner experience of the self as an unattractive social agent, under pressure to limit possible damage via escape or appeasement’ (Gilbert, 2003). Shame often manifests as feelings of being damaged, flawed or socially judged, leading to significant psychological distress (Tangney, 1993). Shame negatively affects both trauma (Saraiya and Lopez-Castro, 2016) and psychosis (Carden et al., 2020; Davies et al., 2025b), and is linked to the development of post-traumatic stress disorder (Budden, 2009; La Bash and Papa, 2014). In psychosis, the presence of shame has been linked to greater emotional distress including heightened depression, anxiety and trauma symptoms (Birchwood et al., 2007; Iqbal et al., 2000; Keen et al., 2017; Turner et al., 2013; Wood and Irons, 2016).

Most research on the connection between shame, trauma and psychosis has focused on bidirectional associations. A limited number of studies have explored the moderating role of shame in the relationship between exposure to potentially traumatic events and psychosis symptoms or compared the individual contributions of shame and trauma to psychotic experiences. Shame has been reported as moderating the association between stressful life events and paranoia (Johnson et al., 2014), and predicting distress associated with paranoia (Carvalho et al., 2016). Despite evidence suggesting the unique influence of both shame and trauma on psychosis and recovery, their combined influence, or whether shame acts as a mediator, remains unexplored.

This study aimed to examine clinical correlates of shame in individuals experiencing psychosis and trauma at a tertiary referral service for psychosis. Specifically, the study aimed to assess the prevalence of trauma and levels of shame, explore associations between shame, trauma exposure, psychotic symptoms and clinical characteristics, and investigate whether shame mediates the association between trauma and psychotic symptoms in individuals receiving care at a tertiary referral service for psychosis in New South Wales, Australia.

## Methods

### Study design

A retrospective audit of clinical data was conducted for individuals who accessed the Tertiary Referral Service for Psychosis (TRSP) between September 2020 and August 2024. All data were collected by clinicians through clinical assessments. Ethics approval was provided by the South Eastern Sydney Local Health District HREC (Ref: 2022/ETH02252).

### Participants

Data from all individuals who were assessed by TRSP were accessed under a waiver of consent and stored in an online database, REDCap (Harris et al., 2009). The TRSP is a public mental health service that provides specialist consultation to mental health teams who support individuals with psychosis (Davies et al., 2023a). Criteria for accessing TRSP include having a diagnosed psychotic illness, being supported by a mental health team and providing consent to participate in the assessment process. Individuals referred to the service are often severely unwell (Davies et al., 2023b; Lappin et al., 2022) or unwilling to complete all assessments, which gave rise to some missing data. The waiver of consent was used to ensure a full cohort and to minimise burden upon people accessing the service.

### Measures

#### Clinical Global Impression Schizophrenia scale

Current severity (past 7 days) of psychosis symptoms was assessed by TRSP psychiatrists through clinical interview using the Clinical Global Impression Schizophrenia scale (CGI-SCH) (Haro et al., 2003). The measure comprises five items, rated on a scale of 1–7, with 1 indicating ‘Normal, not ill’ and 7 ‘Among the most severely ill’. Items relate to ‘positive symptoms’, ‘negative symptoms’ and ‘overall severity’.

#### Others as Shamer Scale II

The Others as Shamer Scale II (OAS2) (Matos et al., 2015) assesses external shame, that is, the feeling of shame that arises from beliefs about others’ negative interpretations of them. The revised 8-item version by Matos et al. (2015) (an adaptation of the original 32-item Others as Shamer Scale (Allan et al., 1994)) was selected for its ease of use in clinical settings, and to reduce burden on individuals. The OAS2 asks respondents to indicate how they think others perceive them, on a scale of 0–4, (0: Never, 1: Seldom, 2: Sometimes, 3: Frequently and 4: Almost always). A total score was calculated by summing the items, with a maximum score of 32.

#### Internalised Shame Scale

A revised version of the Internalised Shame Scale (ISS) (Cook, 1994) was developed and utilised to measure internal shame. The full 30-item measure was initially introduced; however, low levels of completion were observed. In line with the OAS2, an abbreviated version was developed (Supplementary Material) that included the 8 items considered to be most relevant to this population, as ascertained by expert clinical review. The 8-item version was associated with a Cronbach’s alpha of 0.844, supporting the internal consistency. As in the OAS2, individuals were asked to rate how often they experience each item on a 5-point (0–4) scale, with a maximum total score of 32. For individuals who completed the full 30-item measure, data for the relevant 8 items were extracted for analysis. Both shame scales (OAS2 and ISS) were introduced into assessments later than the other measures and had a lower completion rate; therefore, the analyses utilising these represent a sub-sample of the total cohort.

#### Trauma and Life Events Checklist

The Trauma and Life Events Checklist (TALE) (Carr et al., 2018) was used to assess historical exposure to potentially traumatic events (PTEs). Respondents indicated (ticked) which of the 20 events they had experienced over their lifetime. Indexes of cumulative trauma exposure were constructed in three categories for analysis using counts of events indicated for each category: ‘adverse childhood events’ (period of separation from caregiver, witnessing domestic violence, childhood emotional neglect, childhood sexual abuse and childhood physical neglect), ‘other lifespan PTEs’ (permanent separation/loss; bullying; emotional abuse, unexpected loss of home, discrimination, physical abuse, victim of violence, unwanted sexual experiences in adulthood, accidents or natural disasters; exposure to war/conflict; and other events) and ‘psychosis-related PTEs’ (distressing psychosis symptoms, risky behaviours while experiencing psychosis, and distressing experiences with mental health treatment or other service). A total score was calculated by counting the number of events the person had experienced. The measure was collected as part of the assessment and self-completed (*N* = 88). Where this was not possible, data were collated from the person’s medical records and in consultation with their referring team (*N* = 26). Items were recorded ‘yes’ where an event was documented by the person’s treating team, or where the client had reported it in assessments outside of the TALE. Items were recorded as ‘not known’ if there was no mention of their occurrence.

#### Clinical characteristics

Demographic characteristics (i.e. age, sex, cultural background) and clinical characteristics (i.e. clinical diagnosis, duration of illness, co-occurring mental illnesses) were collected. Current mental distress was measured via the Kessler Psychological Distress Scale (K10) (Kessler et al., 2002). Suicidal ideation was measured via a screening tool that included questions on lifetime suicide ideation and attempts, and those occurring in the past 6 months. Current socio-occupational functioning was assessed by the Social and Occupational Functioning Assessment Scale (SOFAS), Health of Nation Outcome Scales (HoNOS) (Wing et al., 1998) and Life Skills Profile-16 (LSP-16) (Rosen et al., 1989).

### Data analysis

Only complete measures were included in the analyses. Key demographic and clinical characteristics are reported through the use of unweighted descriptive statistics using frequencies, means and standard deviations. Medians and interquartile range are used where the data were non-parametric. Pearson’s correlations are used to assess associations where data were normally distributed (i.e. between shame, trauma, HoNOS, K10, LSP-16 and SOFAS) and Spearman’s rank when data were not normally distributed (i.e. for associations with CGI, frequency of suicide attempts, or frequency of past 6-month suicide ideation). For dichotomous variables, point bi-serial correlations were performed. All data were analysed in SPSS, version 29.0.2.0 (IBM Corp, 2023).

#### Trauma exposure

The prevalence of trauma was calculated overall (i.e. any PTE) and for each sub-category.

#### Shame (sub-sample)

For the sub-sample who completed shame measures, average shame scores for each scale are reported (i.e. internal shame, external shame). Associations between shame and clinical characteristics are presented separately for the two shame scales.

#### Power analysis

Given the estimated sample size of 100 clients who completed the trauma measure, at a power of 0.8 and a significance level of 0.05, when conducting correlation analysis, we would be able to detect a minimum effect size of 0.27 for a single predictor. We are confident this would allow any clinically meaningful relationships between trauma, shame and clinical correlates to be detected.

#### Post hoc analyses

To examine the influence of trauma on any relationships between shame and clinical characteristics, partial correlations were conducted with total trauma exposure controlled for. Path analyses were planned to examine shame as a mediator between trauma exposure and any clinical correlates; however, the lack of significance between shame and trauma did not permit this.

## Results

During the 48-month period included, 201 individuals completed assessments with the TRSP service and were included. The majority were male (66.7%) with an average age of 40.3 (SD = 13.3) years. The most common diagnosis was schizophrenia (Table 1), with individuals having experienced psychotic illness for a long duration of time (Mdn: 17 years, IQR: 8–28). Overall severity of psychosis on the CGI indicated high illness severity (Mdn: 5 on 7 item CGI) (Haro et al., 2003). The group also reported high psychological distress (*M* = 24.1, SD = 9.1), and lifetime suicide attempts were reported by almost half (45.0%).

**Table 1.** Demographic and clinical characteristics of the group (*n* = 201).

	Mean	SD
Age (years)	40.3	13.3
	%	n
Sex (% male)	66.7	134
Aboriginal and Torres Strait Islander	10.9	22
Cultural identity (% CALD)	22.9	46
Schizophrenia Dx	62.2	125
Schizoaffective disorder Dx	27.9	56
Other psychotic illness Dx	10.0	20
	Median	IQR
Duration of psychotic illness (years)	17.0	8.0–28.0
Age of psychosis onset (years)	20.0	17.0–25.0
Severity of symptoms (CGI)	5.0	4.0–6.0
Positive symptoms	5.0	4.0–6.0
Negative symptoms	4.0	3.0–5.0
Social and occupational functioning		
SOFAS	35.0	30.0–45.0
HoNOS	28.0	24.0–32.0
LSP-16	28.0	22.0–34.0
	Mean	SD
Mental distress (K10)	24.1	9.1
Anxiety (K10 subscale)	8.9	3.8
Depression (K10 subscale)	15.1	6.2
Internal shame (ISS)	12.5	8.1
External shame (OAS)	11.9	9.6
	%	N
Lifetime suicide attempt (% yes)	44.8	90
Past 6-month suicide ideation (% yes)	24.5	49

### Prevalence of potentially traumatic events

**Figure 1.**
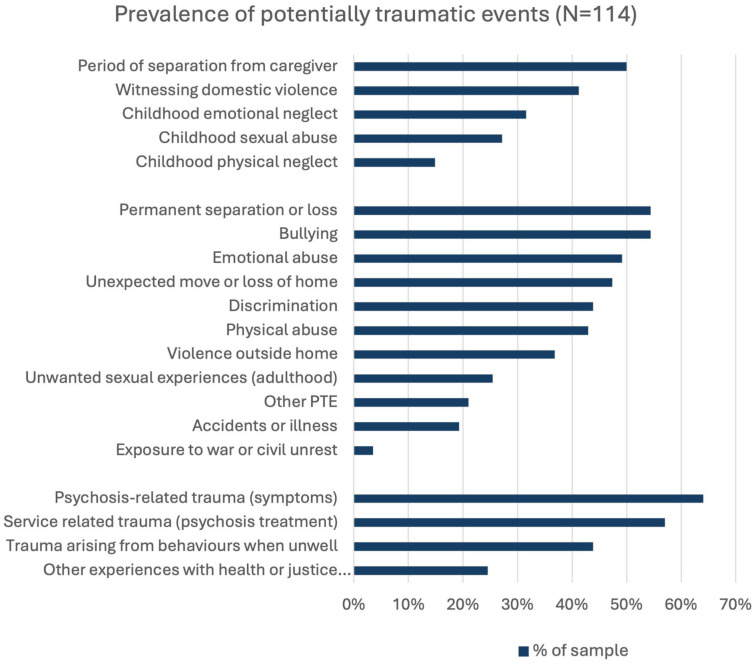
Prevalence of trauma type.

A total of 114 clients completed the trauma measure (57% and 43% missing). Reasons for missing data included the measure being introduced at a later timepoint, illness severity and client refusal. The prevalence of trauma in the group was high (see Figure 1 and Table 2). Of those who completed the measure all reported experiencing at least one potentially traumatic event, with a median of 7 events (IQR: 4–11) (see Figure 1 and Table 2). The most prevalent adverse childhood events reported were ‘a period of separation from a caregiver’ (50.0%) followed by ‘witnessing domestic violence’ (41.2%) (Figure 1). The most commonly reported other lifespan events were ‘permanent separation or loss from a loved one’ (54.4%), and ‘bullying’ (54.4%). Most individuals reported experiencing at least one psychosis-related PTE (74.6%).

**Table 2.** Prevalence of trauma groupings.

	Prevalence of events in group		Median number of events per person	
Trauma category	%	n	Mdn	IQR
Any potentially traumatic event (%)	100.0	114	7	4–11
Adverse childhood events	72.8	83	2	1–3
Other lifespan PTEs events^a^	94.7	108	4	2–7
Psychosis-related PTEs^b^	74.6	85	2	1–3

Trauma measure completed on *n* = 114.

a Not specific to childhood.

b Include only those who completed the measure face-to-face.

### Clinical correlates of trauma exposure

The clinical associations between total trauma exposure and subgroupings of trauma exposure with the other measures and sample characteristics are presented in Table 3. The index of total trauma exposure (r(112) = −0.27, *p* = 0.004) and the ‘other lifespan PTEs’ sub-category (r(108) = −0.27, *p* = 0.005) were significantly and negatively associated with the overall severity of psychosis symptoms. This association was particularly noteworthy for negative symptoms with higher negative symptoms of psychosis associated with lower exposure to trauma (total trauma exposure (*r*(112) = −0.32, *p* = 0.004); other lifespan PTEs (r(108) = −0.35, *p* < 0.001)).

**Table 3.** Associations between trauma exposure and shame with clinical characteristics and outcomes.

	ISS		OAS2		CGI overall		CGI positive		CGI negative		K10		Suicide ideation frequency (past 6 months)		Lifetime suicide (no. of attempts)		HoNOS		SOFAS		LSP-16	
	r	N	r	N	r	N	r	N	r	N	r	N	r	N	r	N	r	N	r	N	r	N
**Trauma**																						
Total PTEs	.151	45	.112	48	–.269**	114	–.186*	114	–.322**	114	.142	80	–.017	100	.202*	96	.094	99	.101	114	.031	95
Adverse childhood PTEs	.100	41	–.035	44	–.060	106	–.082	106	–.153	106	.100	72	.038	92	.123	89	.123	94	.017	106	–.007	90
Other Lifespan PTEs	.145	42	–.007	44	–.268**	110	–.159	110	–.350**	110	.099	76	–.054	96	.217*	92	.185	96	.069	110	.079	92
Psychosis–related PTEs	.058	34	.162	35	–.131	92	–.080	92	–.168	92	.132	69	–.241*	82	.095	80	.054	82	.0.67	92	0.36	79
**Shame**																						
ISS			.763**	49	.167	56	–.062	56	–.002	56	.606**	49	.451**	52	.048	52	.105	43	0.43	56	–.210	41
OAS2					.130	58	.089	58	.022	58	.585**	52	.455**	53	.028	52	–.091	45	.010	58	–.251	42

CGI: Clinical Global Impressions – Schizophrenia, HoNOS: Health of Nation Outcome Scale, ISS: Internal Shame Scale, K10: Kessler Psychological Distress Scale, LSP-16: Life Skills Profile-16, OAS2: Others as Shamer Scale II, SOFAS: Social and Occupational Functioning Assessment Scale.

* *p* < 0.05; **p < 0.005.

There was a significant positive association between the number of trauma events endorsed (total number of trauma events endorsed, *r*(94) = 0.20, *p* = 0.048; number of ‘other lifespan PTEs’, *r*(90) = .22, *p* = .038) and the number of reported lifetime suicide attempts. The number of psychosis-related PTE’s was inversely associated with suicidal ideation frequency across the previous 6-month period, *r*(80) = −0.24, *p* = 0.029.

### Clinical correlates of shame (sub-sample)

A sub-sample of 65 individuals completed at least one shame measure (32.3% and 67.7% missing). Fifty-six individuals (27.9% and 72.1% missing) completed the internal shame measure (ISS) and 58 (28.9% and 71.1% missing) completed the external shame measures (OAS). Missing data were largely due to the measure being introduced later (maximum potential number of clients, *N* = 148). Client illness severity and refusal also led to missing data. A comparison of the sub-sample and those that did not complete the shame measure found they did not differ on demographic characteristics or prevalence of trauma; however, those who did not complete the shame measure had significantly poorer functioning noted on the LSP-16 and SOFAS (Supplementary Material). In the shame sub-sample, psychological distress was found to be significantly and positively associated with internal (*r*(47) = .061, *p* < 0.001) and external (*r*(50) = 0.59, *p* < 0.001) measures of shame.

Higher internal shame (*r*(50) = 0.45, *p* < 0.001) and higher external shame (*r*(51) = 0.46, *p* < 0.001) were both significantly related to increased frequency of suicidal ideation across the previous 6 months. The association with frequency of suicidal ideation remained when total trauma exposure was controlled for, for both internal shame (*r*(39) = 0.44, *p* = 0.003) and external shame (*r*(41) = 0.38, *p* = 0.011).

### Association between trauma and shame

There was no evidence of a significant association between total trauma exposure and either the internal or external shame measures in the sub-sample who completed both measures (*n* = 65), a finding that held across the sub-categories of PTE’s.

### Relationships with suicidal behaviour

Lifetime suicide attempts and past 6-month frequency of suicidal ideation were strongly correlated, *r*(140) = 0.49, *p* < 0.001. A greater number of lifetime suicide attempts, *r*(106) = 0.22, *p* = 0.025, and a higher frequency of suicidal ideation, *r*(102) = 0.49, *p* < 0.001, were also strongly associated with current psychological distress.

### Sensitivity analysis

To examine whether the inclusion of trauma exposure data based on case notes biased the data (i.e. certain items may be underreported), sensitivity analyses were conducted. First, the rates of trauma reported between the methods of data collection were compared, highlighting different patterns in prevalence (Supplementary Material), mainly that few of the case notes included data on psychosis-related trauma. The correlation analyses were then re-run i) with the case note data excluded and ii) by including only the items from the TALE measure that had a similar proportion of reporting between the two methods. This did not change the pattern of significance, and only marginally changed the magnitude of the relationship (Supplementary Material). Given the reduction in sample size when excluding data, it was considered more appropriate to use the full dataset.

## Discussion

This study provides new information about the clinical correlates of shame in individuals experiencing psychosis and builds on previous work examining the influence of trauma on mental health outcomes. It is, as far as the published literature indicates, the first to identify shame as a correlate of suicidal ideation in this population, offering potentially important insights for clinical approaches to suicide risk assessment.

The prevalence of trauma was high, and the participants who were able to complete the trauma scale all reported experiencing at least one potentially traumatic event (PTE). In line with other studies in the field (Bendall et al., 2008; Duhig et al., 2015), almost three-quarters of the group reported adverse childhood events, with almost a third reporting childhood sexual abuse. In addition, most reported psychosis-related trauma. Of concern, 57% of the responses indicated that such trauma was associated with mental health care and treatment. Other researchers have suggested trauma related to mental health care may arise from the use of restrictive and coercive practices resulting in feelings of distrust, shame and helplessness (Lu et al., 2017). With services increasingly adopting trauma-informed models based on awareness of the prevalence of trauma among populations accessing care, it is also crucial that trauma which occurs in care and treatment is directly considered and addressed. Incorporating ‘shame-sensitive practice’ alongside trauma-informed approaches may also provide opportunities to acknowledge, address and minimise shame (Dolezal and Gibson, 2022).

Consistent with previous research, there was a positive relationship between the cumulative exposure to PTEs and the number of lifetime suicide attempts, indicating that those who reported a higher number of lifespan PTEs also reported a higher frequency of suicide attempts (Dube et al., 2001; McFeeters et al., 2015), reinforcing the need for suicide treatment and support to not only focus on assessment of risk but also to help build resilience for facing future events (Sher, 2019). For example, psychosocial interventions that help to develop strategies to manage emotional distress may be beneficial (Phalen et al., 2024).

The finding that current shame is associated with recent frequency of suicidal ideation is noteworthy. Psychological distress is a known risk factor for suicidal behaviour (Chamberlain et al., 2009) and has also been linked to shame (Tangney, 1993). Here, shame and psychological distress were strongly associated, which may suggest a path through which shame could exert an influence on suicidality; however, larger-scale studies are needed to develop robust path models that allow testing of this and to aid understanding the direction of effect. Shame has also been found to drive social withdrawal (Tangney et al., 1992). The presence of shame may therefore be a contributing and compounding factor for social isolation, a recognised mediator between psychosis and suicide (Bornheimer et al., 2020). Furthermore, in McCarthy-Jones (2017) model of the trauma-psychosis association, shame is proposed to mediate the relationship through the adoption of avoidant and absorbed coping strategies (e.g. rumination). Whether these coping strategies have additional impacts on other mental health outcomes, in the context of shame, has not yet been explored. Current suicidal ideation is a strong predictor of suicide attempts (Large et al., 2020); thus, the identification of shame as a potential modifiable factor could have important implications for clinical practice; however, more information is needed on the direction of the relationship and sources of shame. Research exploring individuals’ subjective experiences of shame may provide more detailed insight into the relationship between shame and psychosis. Future work could also examine whether reducing shame through psychological interventions can influence suicidal ideation (Heriot-Maitland et al., 2023).

In contrast to previous research (Bailey et al., 2018), psychosis symptom severity was negatively associated with trauma. One explanation may be the cohort studied here: individuals who reported greater exposure to PTE’s may have been more likely to be referred to the tertiary service for support for reasons other than psychosis symptom severity, for instance, ongoing distress and impaired function potentially arising from adverse experiences. Furthermore, the current literature on trauma suggests that it is the meaning applied to an event that more strongly influences whether it leads to a range of distressing outcomes, rather than the event itself (Johnstone and Boyle, 2018). Exposure to an increasing number of PTEs would therefore not necessarily lead to increased severity of symptoms or distress. This may also explain the lack of association between trauma and other measures, including shame, in this cohort (i.e. more events do not equate to higher shame, high shame can be experienced for just one event). This highlights the need to go beyond a checklist approach to screening for trauma and instead to ensure that trauma-informed approaches explore how adverse events have personally affected individuals (Blue Knot Foundation, 2012). A further possible explanation is that the negative associations between total PTEs and symptom severity, and between other lifespan PTEs and symptom severity, were driven by a reduced expression of negative symptoms contributing to lower total symptom severity.

A strength of this study is the use of real-world clinical data. However, due to the nature of data, there are also limitations. First, the sample with all relevant measures completed was small, which may have led to some correlations being underpowered. For instance, the relationship between PTE’s and shame scales were of moderate size but did not reach significance, so planned mediation analyses could not be conducted. Second, there may have been sampling bias due to self-selection. That is, individuals with higher levels of shame may have been more likely to refuse completion of the measure to avoid uncomfortable or confronting questions. In addition, individuals experiencing the most severe psychosis symptoms and deemed not to have capacity, were not asked to complete the shame or trauma measure, which may have influenced the data. For instance, the extraction of data on trauma exposure from medical records may have led to lower prevalence rates reported here, given the underreporting in clinical settings (Neill and Read, 2022). Similarly, on average, the current sample had a long duration of experiencing psychosis; therefore, studies with individuals experiencing a first-episode psychosis are needed to explore whether the same relationships are observed across the spectrum.

Finally, the findings are correlational and based on assessment at one timepoint; therefore, it is not possible to ascertain the direction of effects or more detail on the nature of relationships. More information is needed on the sources of shame in this group, including whether it is associated with psychosis, trauma or other factors. Prospective studies could provide more information on whether the relationships noted here are sustained over time.

## Conclusions

These findings highlight associations between suicidal behaviour with trauma exposure and shame. While exposure to PTEs was associated with lifetime suicide attempts, shame was more strongly related to current suicidal ideation and psychological distress. Although shame has been linked to suicidality in the general population, this finding has not previously been reported in populations with experiences of psychosis and warrants further investigation. Future work could examine whether interventions that target shame have any effect on suicide ideation, while studies with larger samples could be used to develop robust path models to better understand how shame influences suicide ideation.

## Supplemental Material

sj-docx-1-anp-10.1177_00048674251411085 – Supplemental material for Shame and trauma are critical to understanding the impacts of psychosis: Examining clinical correlates within a tertiary psychosis service cohort Supplemental material, sj-docx-1-anp-10.1177_00048674251411085 for Shame and trauma are critical to understanding the impacts of psychosis: Examining clinical correlates within a tertiary psychosis service cohort by Kimberley Davies, Julia M Lappin, Sophie Isobel and Zachary Steel in Australian & New Zealand Journal of Psychiatry
