# Supplementary material for: Shame and trauma are critical to understanding the impacts of psychosis: Examining clinical correlates within a tertiary psychosis service cohort
Source: Aust N Z J Psychiatry. 2026 Jan 27;60(6):553–61. doi: 10.1177/00048674251411085 (PMC13191054; doi:10.1177/00048674251411085)
Supplement: sj-docx-1-anp-10.1177_00048674251411085 – Supplemental material for Shame and trauma are critical to understanding the impacts of psychosis: Examining clinical correlates within a tertiary psychosis service cohort [file sj-docx-1-anp-10.1177_00048674251411085.docx]

**Supplementary Material**

# Internalised Shame Scale (adapted from Cook, 1990)

Directions: Below is a list of statements describing feelings or experiences that you may have from time to time or that are familiar to you because you have had these feelings and experiences for a long time. Most of these statements describe feelings and experiences that are generally painful or negative in some way. Some people will seldom or never have had many of these feelings. Everyone had had some of these feelings some of the time, but if you find that these statements describe the way you feel a good deal of the time, it can be painful just reading them. Try to be as honest as you can in responding.

| 0 | 1 | 2 | 3 | 4 |
| --- | --- | --- | --- | --- |
| Never | Seldom | Sometimes | Often | Almost always |

Read each statement carefully and circle the number to the left of the item that indicates the frequency with which you find yourself feeling or experiencing what is described in the statement. Use the scale below. DO NOT OMIT ANY ITEM.

| 1. I feel like I am never quite good enough. | 0 | 1 | 2 | 3 | 4 |
| --- | --- | --- | --- | --- | --- |
| 2. I think that people look down on me. | 0 | 1 | 2 | 3 | 4 |
| 3. I see myself as being very small and insignificant. | 0 | 1 | 2 | 3 | 4 |
| 4. I feel intensely inadequate and full of self-doubt. | 0 | 1 | 2 | 3 | 4 |
| 5. I feel as if I am somehow defective as a person, like there is something basically wrong with me | 0 | 1 | 2 | 3 | 4 |
| 6. I have an overpowering dread that my faults will be revealed in front of others. | 0 | 1 | 2 | 3 | 4 |
| 7. I would like to shrink away when I make a mistake. | 0 | 1 | 2 | 3 | 4 |
| 8. I feel I am a person of worth at least on an equal plane with others. | 0 | 1 | 2 | 3 | 4 |

Correlation matrix with those who completed TALE by case notes excluded

|  | ISS | OAS2 | CGI | K10 | Suicide ideation frequency | Past 6-month suicide | Lifetime suicide | HoNOS | SOFAS | LSP-16 |
| --- | --- | --- | --- | --- | --- | --- | --- | --- | --- | --- |
| Total PTEs | .185  N=29 | -.030  N=31 | -.338**  N=84 | .147  N=65 | -.042  N=77 | -.062  N=77 | .292*  N=74 | .209  N=77 | .081  N=84 | .018  N=74 |
| Childhood PTEs | -.004  N=29 | -.140  N=31 | -.293**  N=83 | .113  N=65 | .016  N=76 | -.022  N=76 | .203  N=73 | .116  N=76 | .078  N=83 | -.067  N=73 |
| Lifetime PTEs | .251  N=29 | -.010  N=31 | -.316**  N=84 | .116  N=65 | -.020  N=77 | -.059  N=77 | .294*  N=74 | .248*  N=77 | .040  N=84 | .057  N=74 |
| Psychosis-related PTEs | .060  N=29 | .123  N=31 | -.122  N=82 | .149  N=64 | -.196  N=76 | -.110  N=76 | .103  N=73 | .002  N=76 | .117  N=82 | -.044  N=73 |

Correlation matrix for abbreviated (5-item) TALE

|  | ISS | OAS2 | CGI | K10 | Suicide ideation frequency | Lifetime suicide | HoNOS | SOFAS | LSP-16 |
| --- | --- | --- | --- | --- | --- | --- | --- | --- | --- |
| Full TALE | .223  N=42 | .127  N=45 | -.371**  N=106 | .161  N=75 | -.029  N=94 | .241*  N=90 | .084  N=94 | .127  N=106 | .003  N=90 |
| Abbrev TALE | .080  N=25 | -.089  N=26 | -.265*  N=66 | .045  N=52 | .126  N=63 | .383**  N=62 | .286*  N=61 | -.025  N=66 | .111  N=58 |

Prevalence of PTE types using face-to-face versus case note data

|  | No shame measure | | Shame sub sample | |  |
| --- | --- | --- | --- | --- | --- |
|  | Mean | SD | Mean | SD |  |
| Age (years) (M, SD) | 40.8 | 13.6 | 39.2 | 12.5 | P= .47 |
|  |  |  |  |  |  |
|  | % | n | % | n |  |
| Sex (% male) | 65.4 | 89 | 69.2 | 45 | P=.59 |
| Aboriginal and Torres Strait Islander | 11.8 | 16 | 9.2 | 6 | P=.67 |
| Cultural identity (% CALD) | 22.8 | 31 | 23.1 | 15 | P=.67 |
| Schizophrenia Dx | 56.6 | 77 | 73.8 | 48 | P=.06 |
| Schizoaffective disorder Dx | 33.1 | 45 | 16.9 | 11 | P=.06 |
| Other psychotic illness Dx | 10.3 | 14 | 9.2 | 65.0 | P=.06 |
|  |  |  |  |  |  |
|  | Median | IQR | Median | IQR |  |
| Duration of psychotic illness (years) | 18.0 | 8.0 – 29.0 | 14.0 | 7.0 – 27.0 | P=.38 |
| Age of psychosis onset (years) | 19.0 | 17.0 – 25.0 | 20.0 | 17.0 – 25.0 | P=.62 |
| Severity of symptoms (CGI) | 5.0 | 4.0 – 6.0 | 5.0 | 4.0 – 6.0 | P=.94 |
| Positive symptoms | 5.0 | 4.0 – 6.0 | 5.0 | 4.0 – 6.0 | P=.89 |
| Negative symptoms | 4.0 | 3.0 – 5.0 | 4.0 | 3.0 – 4.0 | P=.25 |
| Social and occupational functioning |  |  |  |  |  |
| SOFAS | 35.0 | 29.0 – 40.0 | 35.0 | 30.0 – 45.0 | P=.65 |
| HoNOS | 29.0 | 25.0 – 33.0 | 25.0 | 21.0 – 28.0 | P<.001 |
| LSP-16 | 29.0 | 24.0 – 35.0 | 23.0 | 17.0 – 30.0 | P<.01 |
|  |  |  |  |  |  |
|  | Mean | SD | Mean | SD |  |
| Mental distress (K10) | 24.1 | 9.6 | 24.1 | 8.6 | P=.15 |
| Anxiety (K10 subscale) | 8.8 | 4.0 | 9.0 | 3.7 | P=.42 |
| Depression (K10 subscale) | 15.1 | 6.4 | 15.1 | 6.0 | P=.38 |
|  | % | N | % | N |  |
| Lifetime suicide attempt (% yes) | 39.7 | 54 | 55.4 | 36 | P=.97 |
| Past 6-month suicide ideation (% yes) | 22.1 | 30 | 29.2 | 19 | P=.39 |
| Trauma | % | N = 62 | % | N = 48 |  |
| Any potentially traumatic event (%) | 100.0 | 62 | 100.0 | 48 |  |
| Adverse childhood events | 72.6 | 45 | 79.2 | 38 |  |
| Other lifespan PTEs events^$^ | 96.8 | 60 | 100.0 | 48 |  |
| Psychosis-related PTEs^1^ | 79.0 | 49 | 75.0 | 36 |  |
